# Isobavachalcone Attenuates MPTP-Induced Parkinson's Disease in Mice by Inhibition of Microglial Activation through NF-κB Pathway

**DOI:** 10.1371/journal.pone.0169560

**Published:** 2017-01-06

**Authors:** Haoran Jing, Shaoxia Wang, Min Wang, Wenliang Fu, Chao Zhang, Donggang Xu

**Affiliations:** 1 Laboratory of Genome Engineering, The Beijing Institute of Basic Medical Sciences, Beijing, China; 2 Tianjin Key Laboratory of Chinese Medicine Pharmacology, Tianjin University of Traditional Chinese Medicine, Tianjin, People's Republic of China; Georgia Regents University, UNITED STATES

## Abstract

Parkinson's disease (PD) is a complex multi-system and age-related neurodegenerative disorder. The intervention targeting neuroinflammation in PD patients is one effective strategy to slow down or inhibit disease progression. Microglia-mediated inflammatory response plays an important role in Parkinson's, Alzheimer's and other cerebral diseases. Isobavachalcone is a main component of Chinese herb medicine *Psoralea corylifolia*, which function includes immunoregulation, anti-oxidation and the regulation of β-amyloid (Aβ42) deposited in hippocampus in Alzheimer's patients. Whether it has the therapeutic effect on Parkinson's disease, however, is unclear. In this study, we found that isobavachalcone could effectively remit Parkinson's disease induced by 1-methyl-4-phenyl-1,2,3,6- tetrahydropyridine (MPTP), prolong the residence time of mice on Rota-rod and alleviate the neuronal necrosis. It also inhibited the over-activation of microglia, and decreased the expression of IL-6 and IL-1β in the brain of PD mice. In vitro, isobavachalcone could inhibit nuclear factor-kappaB (NF-κB) pathway through inhibiting the LPS-induced transfer of NF-κB subunit from cytoplasm to nucleus in BV-2 cells. Isobavachalcone decreased the LPS-induced oxidative stress and the expression of inflammatory cytokines, and provided a neuroprotective effect by antagonizing microglia-mediated inflammation. Our results indicated that isobavachalcone may be a candidated drug against Parkinson's disease with great clinical potential.

## Introduction

Microglia are the resident immune cells in the brain. Many studies have consistently demonstrated that the microglia-mediated inflammatory response plays an important role in cerebral ischemia, Alzheimer's disease, Parkinson's disease, and other cerebral pathological processes. Microglia are the potential target of therapeutic intervention to inhibit both the hyperactive microglia-induced inflammation and its secondary injury in these diseases.

At present, many natural products extracted from plants show the inhibitory effect on microglia activation, such as *Fructus psoraleae* extracted from the dried fruits of leguminous plant. *Fructus psoraleae* mainly consists of coumarin and prenylflavone derivatives, which presents versatile effects including anti-bacterial [1], anti-oxidation [2], anti-tumor [3, 4], the inhibition of platelet aggregation [5], immunoregulation, hormonal regulation [6], and bone strengthening [7], etc. Chen et al’ reported that isobavachalcone, as an active ingredient in *Fructus psoraleae*, could regulate the aggregation process of β-amyloid (Aβ42) [8].

To investigate the role of isobavachalcone in the PD, a PD model in mouse was established by intraperitoneal injection of MPTP (1-methyl-4-phenyl-1,2,3,6- tetrahydropyridine). The effect of isobavachalcone on neuronal injury and microglia activation was observed, and the expression of inflammatory cytokines before and after administration was detected. Furthermore, a mouse microglia cell line (BV-2 cells) was stimulated by lipopolysaccharide (LPS) to establish a nerve inflammatory cell model for exploring the effect of isobavachalcone on activation of microglia and its mechanism. Our studies may provide the basis for the clinical applications of isobavachalcone against Parkinson's diseases.

## Materials and Methods

### Cell cultures

Mouse microglia (BV-2 cells) and neurocytoma cells (Neuro-2a cells) were purchased from Institute of Basic Medical Science, Chinese Academy of Medical Sciences.

#### BV-2 cells

The mouse microglial cell line BV-2 was purchased from Institute of Basic Medical Science, Chinese Academy of Medical Sciences. BV-2 was cultured in DMEM medium (Gibco Life Tech, Cat.11995 USA), supplemented with 0.1% penicillin-streptomycin (Invitrogen) and 10% foetal bovine serum (FBS, Invitrogen). Cells were maintained at 37°C in a 5% CO_2_ humidified atmosphere.

#### Neuro-2a cells

The mouse neurocytoma cell line Neuro-2a was purchased from Institute of Basic Medical Science, Chinese Academy of Medical Sciences. Neuro-2a was cultured in MEM medium (Gibco Life Tech, Cat.41500034, USA), supplemented with 0.1% penicillin-streptomycin (Invitrogen) and 10% foetal bovine serum (FBS, Invitrogen). Cells were maintained at 37°C in a 5% CO2 humidified atmosphere.

### Experimental animals

Mice used in the animal experiment were provided by Vital River Experimental Technology Company Ltd., Beijing. Forty-eight male C57BL/6 mice with an average body weight of 22–25 g were randomly divided into 4 groups: control, Parkinson's model group, and 2 isobavachalcone (Tianjin Zhongxin Pharmaceutical Group Co., China, HPLC ≥98%) treatment groups each with 12 mice.

### Animal experimental protocol

At day 1 after pre-administration (i.g.), MPTP (20mg/kg, Sigma,USA) was injected for 4 times with 2 h intervals. The administration of isobavachalcone by gavage was performed at the time point of 1 h after the first MPTP injection. Isobavachalcone at the dose of 50 and 10 mg/kg/day was administered for 7 days respectively, then the animals were sacrificed and their blood was sampled. The whole brain removed from 6 mice per group was dissected after heart perfusion with physiological saline and 4% paraformaldehyd, and then fixed in 4% paraformaldehyd for immunofluorescence and immunohistochemical assays. The ventral mid-brain and corpus striatum from the mice without heart perfusion were snap-frozen in liquid nitrogen and stored at −80°C for biochemical analysis. The study protocol was reviewed and approved by the Ethics Committee of Institute of Basic Medical Sciences in Beijing, China.

### Test of motor, balance and coordination abilities

Rota-rod was used to test the passive motor ability of each mouse. The duration for a mouse to stay on the rod rolling at 10 rpm was recorded to assess its motor coordination ability.

### Immunofluorescence and immunohistochemistry assays

Frozen sectioning was performed on the brain tissues along the sagittal plane. Anti-Tyrosine Hydroxylase antibody (TH, dopaminergic tyrosine hydroxylase immunopositive neurons, Abcam, USA) was used for immunofluorescence test. Anti-Iba1 antibody (Microglia specific calcium binding protein, microgliosis, Abcam,USA) and an antibody of glial fibrillary acidic protein (GFAP, Astrocyte specific protein, marker of astrogliosis, CST,USA) were used for immunohistochemical (IHC) staining. A laser scanning confocal microscope and Olympus microscope were used to observe the positive cells, and the positive cells of each section were recorded and calculated from five random visual fields.

### Total RNA extraction and real-time quantitative PCR

The mRNAs were extracted by RNA Pure Kit (TIANGEN, China) for animal tissue total RNA extraction (spin column), and then used as template for cDNAs synthesized by RT-PCR. The primers for RT-PCR were listed in Table 1.

**Table 1:** The primers for real-time PCR.

Gene(Mouse)	Forward Primer (5’-3’)	Reverse Primer (5’-3’)
GAPDH	CTTCACCACCATGGAGAAGGC	GGCATGGACTGTGGTCATGAG
iNOS	GGCAGCCTGTGAGACCTTTG	GCATTGGAAGTGAAGCGTTTC
TNFα	CGGGGTGATCGGTCCCCAAAG	GGAGGGCGTTGGCGCGCTGG
IL-6	CCAGAGATACAAAGAAATGATGG	ACTCCAGAAGACCAGAGGAAA
IL-1β	CGCAGCAGCACATCAACAAGAGC	TGTCCTCATCCTGGAAGGTCCACG
IL-10	CAGAGCCACATGCTCCTAGA	GTCCAGCTGGTCCTTTGTTT

BV-2 cells were treated with LPS (the final concentration: 0.1 μg/ml) or LPS plus isobavachalcone (the final concentration: 5 μM) for 8 h, from which mRNA was extracted using Trizol (Invitrogen,USA). The primers for real-time PCR were listed in Table 1.

### Determination of the serum levels of NO, TNF-α and IL-6

BV-2 cells were treated with LPS (the final concentration: 0.1 μg/ml) or LPS plus isobavachalcone (the final concentration: 0.1, 1, 10, 20 μM) for 24 h. Griess method was used to determine NO concentration; the concentrations of TNF-α and IL-6 were determined using ELISA kits (R&D system, USA).

### Determination of NF-κB activity

BV-2 cells were treated with LPS (the final concentration: 0.1 μg/ml) or LPS plus isobavachalcone (the final concentration: 5 μM) for 45min. Then the nucleoprotein was extracted, and the transcriptional activity of p65 was detected by Transcription Factor Assay Kit (Active Motif #400098 NF-κB p65 EZ-TFA).

### Western blot

Brain tissue was homogenized (1:8, W/V) on ice with a microcontent motor-operated tissue homogenizer in ice-cold lysis buffer (1×PBS, 1% Nonidet P-40, 0.5% sodium deoxycholate, and 0.1% SDS, RIPA) supplemented with protease inhibitors. Lysates were centrifuged at 10,000×g for 15 min at 4°C, the supernatants were collected.

BV-2 cells were treated with LPS (the final concentration: 0.1 μg/ml) or LPS plus isobavachalcone (the final concentration: 5 μM) for 45min. The nucleoprotein was extracted from the cells in each group.

Protein concentrations were determined by a BCA protein assay.and then Western Blot was performed to examine the p65 expression level using McAb to p65 (1:1000; CST, USA). Briefly, equal amounts of protein (30 μg) were separated by SDS-PAGE and electroblotted onto polyvinylidene fluoride (PVDF) membrane (Millipore). After non-specific antibody binding was blocked with 5% non-fat dry milk, membranes were incubated at 4°C overnight with McAb p65 (1:1000; CST, USA) and monoclonal mouse anti-GAPDH (1:2000, CST, USA). After washing in TBST, the immunoblots were incubated with horseradish peroxidase conjugated secondary antibodies (1:8000, CST) for 1 h. The immunoblots were developed with an enhanced chemiluminescence (ECL) reagents (Millipore, USA), and measured with Quantity Software (Bio-Rad, CA).

### Detection of nuclear transfer level of NF-κB p65

BV-2 cells were treated with LPS (the final concentration: 0.1 μg/ml) or LPS plus isobavachalcone (the final concentration: 5 μM) for 45 min, washed for 3 times with PBS, fixed with 4% paraformaldehyde at room temperature (RT) for 20 min, washed 3 times with PBS, treated with PBS solution containing 0.1% TritonX-100 at RT for 10 min, washed 3 times with PBS, and then incubated with 4% BSA solution at 37°C for 30 min to block non-specific reactions. The antibody to NF-κB p65 was added and then stored at 4°C overnight. The 2nd antibody of goat to mouse was added and kept in a darkroom at RT for 1.5 hrs, washed 3 times with PBS, and then the nucleus was counterstained with DAPI (20× diluted). After incubation for 10 min at RT in a darkroom, the samples were rinsed and then observed by means of the laser scanning confocal microscopy.

### Cell viability assay in BV-2 cells

The BV-2 cells were grown in 96-well plate (Corning Life Sciences, USA), and LPS (final concentration 0.1 μg/ml) or LPS with plus isobavachalcone (final concentration 5 μM) was added. Cell viability was assessed using Cell Counting Kit-8(DOJINDO, Japan).

### Effect of isobavachalcone-treated BV-2 cells supernatant on activity of neuro-2a cells

BV-2 cells were treated with LPS (the final concentration: 0.1 μg/ml) or LPS with plus isobavachalcone (the final concentration: 5 μM) for 24 h to prepare conditioned media (CM) for the microglia in 4 groups: ①control (DMEM basic culture medium), ②LPS (supernatant from LPS treated BV-2 cells), ③LPS+Iso (supernatant from LPS plus isobavachalcone treated BV-2 cells), ④LPS/Iso (isobavachalcone (final concentration 5μM) dissolved in supernatant from LPS treated BV-2 cells). Neuro-2a cells were used as the target cells. The above-mentioned CM were separately transferred into Neuro-2a cells (96-well plate). After incubation for 24 hrs, the cytotoxic effect of each CM was examined using Cell Counting Kit-8(DOJINDO, Japan).

### Cytotoxicity assay in a co-culture of neuro-2a and BV-2 cells

The BV-2 cells were co-cultured with neuro-2a cells to study the regulation of neuro-2a survival by the LPS-stimulated microglia. The BV-2 microglial cells were grown in Transwell inserts (pore size, 0.4 μm; Corning Life Sciences, USA), and LPS (final concentration 0.1 μg/ml) or LPS with plus isobavachalcone (final concentration 5 μM) was added. The neuro-2a cells were then transferred onto the inserts containing BV-2 cells. In the Transwell co-culture system, microglial cells communicate with neuro-2a through the semi-permeable membrane without direct cell contact. Cell viability was assessed using Cell Counting Kit-8(DOJINDO, Japan).

### Statistical analysis

Results were presented as the mean±SD Values. Statistical significance was determined using Student’s *t*-test by origin 7.5 software. The results were considered significant when *P*-value was less than 0.05, very significantly different when *P*-value was less than 0.01

## Results

### Effects of isobavachalcone on mouse motor, balance and coordination abilities

**Fig 1:**
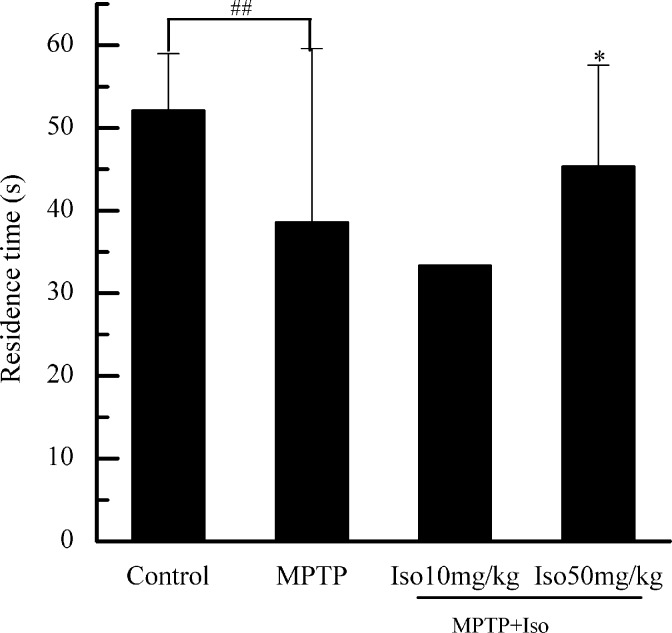
Effects of isobavachalcone on motor, balance and coordination abilities of PD mouse. Isobavachalcone (Iso, 50 mg/kg) significantly prolonged the residence time of mice on Rota-rod. Bars indicate the mean±SD of three independent experiments. ## p<0.01 indicating very significantly different from Control group. * p<0.05 indicating statistically significantly different from MPTP injury group.

The stay time on Rota-rod was significantly reduced in the mice of MPTP group compared with that of control group, confirming that the PD model was successfully established. Moreover, the stay time of isobavachalcone (50 mg/kg)-treatment group was obviously longer than that of model group, which indicated that isobavachalcone improved the motor, balance and coordination abilities of PD mouse (Fig 1).

### Effects of isobavachalcone on microglia, astrocytes and neurons

**Fig 2:**
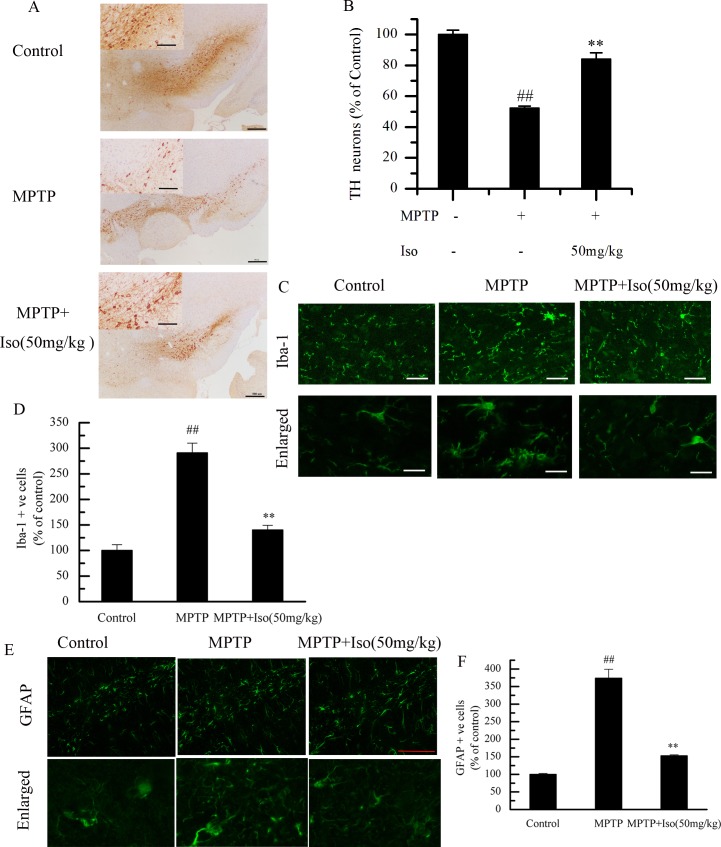
Effects of isobavachalcone on microglia, astrocytes and neurons. (A) Brain sections were immunostained for TH immunoreactivity in SNpc. The activation of microglia and astrocytes respectively were detected by Iba-1 and GFAP immunostaining (figs C and E). The number of TH, Iba-1 and GFAP positive cells were counted, and the data were expressed as mean±SD (figs B, D and F). Those results show that isobavachalcone (Iso, 50 mg/kg) could inhibit either the elevated levels of Iba-1 and GFAP, or the necrosis of neurons. Images were obtained from the SNpc of Iba-1, TH and GFAP. Scale bars: 200μm and 500μm in A, and 50μm in C and E, n = 4. ## p<0.01 indicating very significantly different from Control group. ** p<0.01 indicating very significantly different from MPTP injury group.

As shown in Fig 2C, 2D, 2E and 2F, the levels of IBa-1 and GFAP, as the specific markers of microglia and astrocytes respectively in the model group, were significantly higher than that of control group, which indicated that MPTP increased the activation of microglia and astrocytes. The levels of IBa1 and GFAP in isobavachalcone treated group were dramatically lower than that of model group, suggesting that isobavachalcone could inhibit the activation of microglia and astrocytes. In addition, isobavachalcone significantly inhibited the necrosis of neurons injured by MPTP (Fig 2A and 2B).

### Effects of isobavachalcone on microglia inflammatory cytokines in mouse brain tissues and BV-2 cells

**Fig 3:**
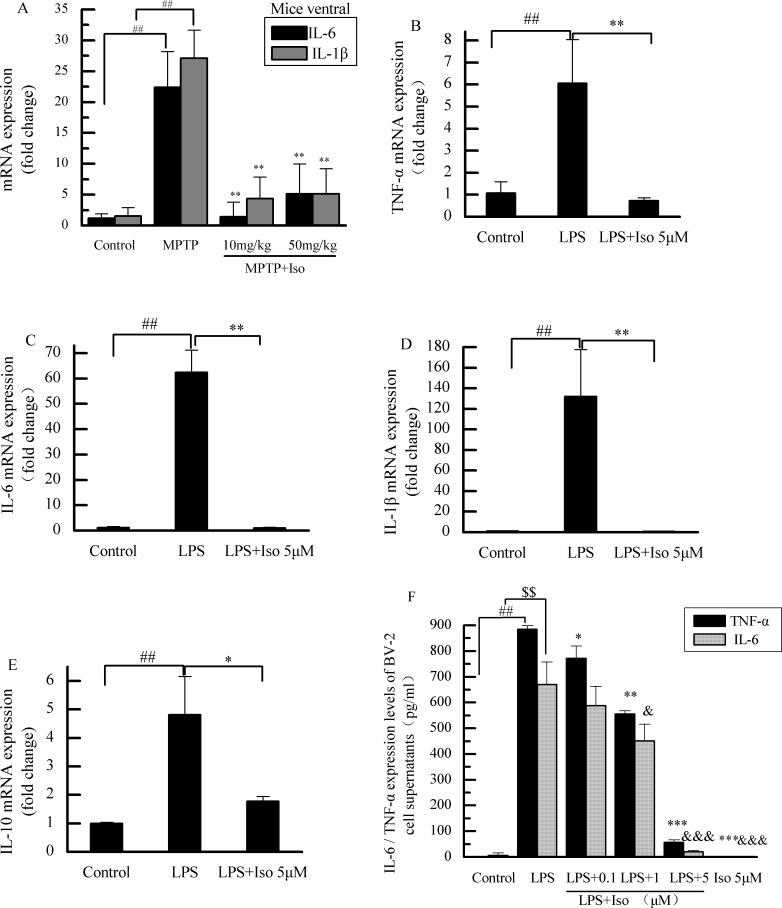
Changes of microglia inflammatory cytokines in vitro and in vivo. **A**: Isobavachalcone (Iso) significantly inhibited the transcription of IL-6 and IL-1β induced by MPTP injury. B-E: Isobavachalcone inhibited the transcriptional levels of TNF-α, IL-6, IL-1β, and IL-10 induced by LPS in BV-2 cells. F: The expression levels of both TNF-α and IL-6 were decreased with the increasing of the administration isobavachalcone concentration. Bars indicate the mean±SD. ##,$ $ p<0.01 vs. respective control group. *,& p<0.05, **p<0.01, ***,&&& p<0.001 vs. PD model or LPS-treated group.

Isobavachalcone could significantly inhibit the transcription of IL-6 and IL-1β in PD mouse (Fig 3A), it also decreased the transcriptional levels of TNF-α, IL-6, IL-1β, and IL-10 in BV-2 cells stimulated by LPS (Fig 3B–3E). Moreover, as shown in Fig 3F, isobavachalcone decreased the expression levels of TNF-αand IL-6 induced by LPS in a dose-dependent manner.

### Effects of isobavachalcone treatment on NO and iNOS

**Fig 4:**
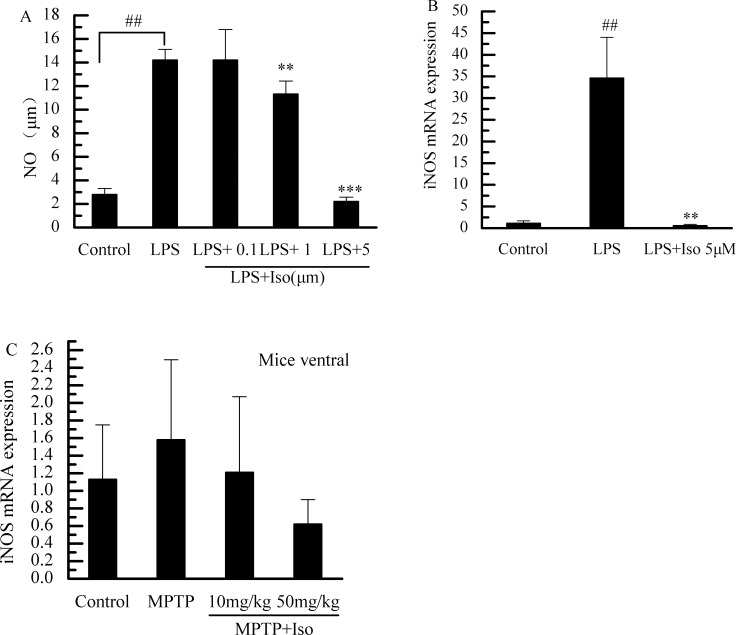
Effects of isobavachalcone treatment on NO and iNOS. (A, B) Isobavachalcone (Iso) significantly inhibited the levels of NO and the transcriptional levels of iNOS in LPS-treated BV-2 cells. C: Effect of isobavachalcone treatment on iNOS mRNA transcriptional levels in PD mouse brain. Bars indicate the mean±SD of three independent experiments. ##p<0.01 vs. Control group. ** p<0.01 and *** p<0.001 vs. LPS-treated group.

Isobavachalcone could inhibit the production of NO in LPS-treated BV-2 cells in a dose-dependent manner (Fig 4A). PCR results showed that it significantly decreased the LPS-induced iNOS transcription in BV-2 cells (Fig 4B), but could not obviously influence the iNOS transcription in mouse mid-brain and substantia nigra (Fig 4C).

### Effect of isobavachalcone on NF-κB pathway

**Fig 5:**
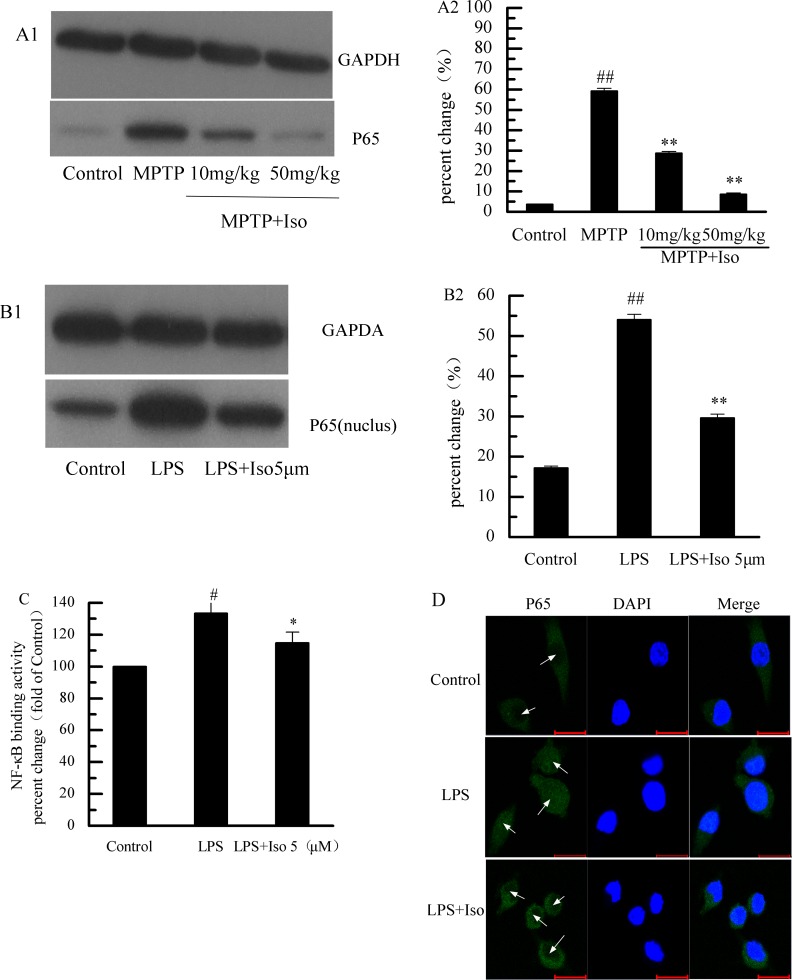
Effect of isobavachalcone on NF-κB pathway. (A1, A2, B1, and B2) The expression levels of p65 in BV-2 cells and the mouse brain tissue. (C and D) Isobavachalcone (Iso) inhibited the DNA binding activity (C) and nuclear transfer (D) of NF-κB p65. Bars indicate the mean±SD of three independent experiments. # p<0.05, ## p<0.01 in comparison to Control group. * p<0.05, ** p<0.01 in comparison to LPS group.

Isobavachalcone inhibited the activation of p65 subunit in brain tissues caused by MPTP injury (Fig 5A1 and 5A2), and also decreased the expression of p65 of the nucleoprotein *in vitro* in BV-2 cells (Fig 5B1 and 5B2). Meanwhile, the results showed that LPS activated the DNA-binding activity of NF-κB transcription factor, which was significantly prohibited by isobavachalcone (Fig 5C). As shown by laser scanning confocal microscopy, LPS induced the transfer of p65 subunit from cytoplasm to nuclus, whereas isobavachalcone blocked this process (Fig 5D).

### Effects of isobavachalcone treatment on BV-2 cells and neuro-2a cells

**Fig 6:**
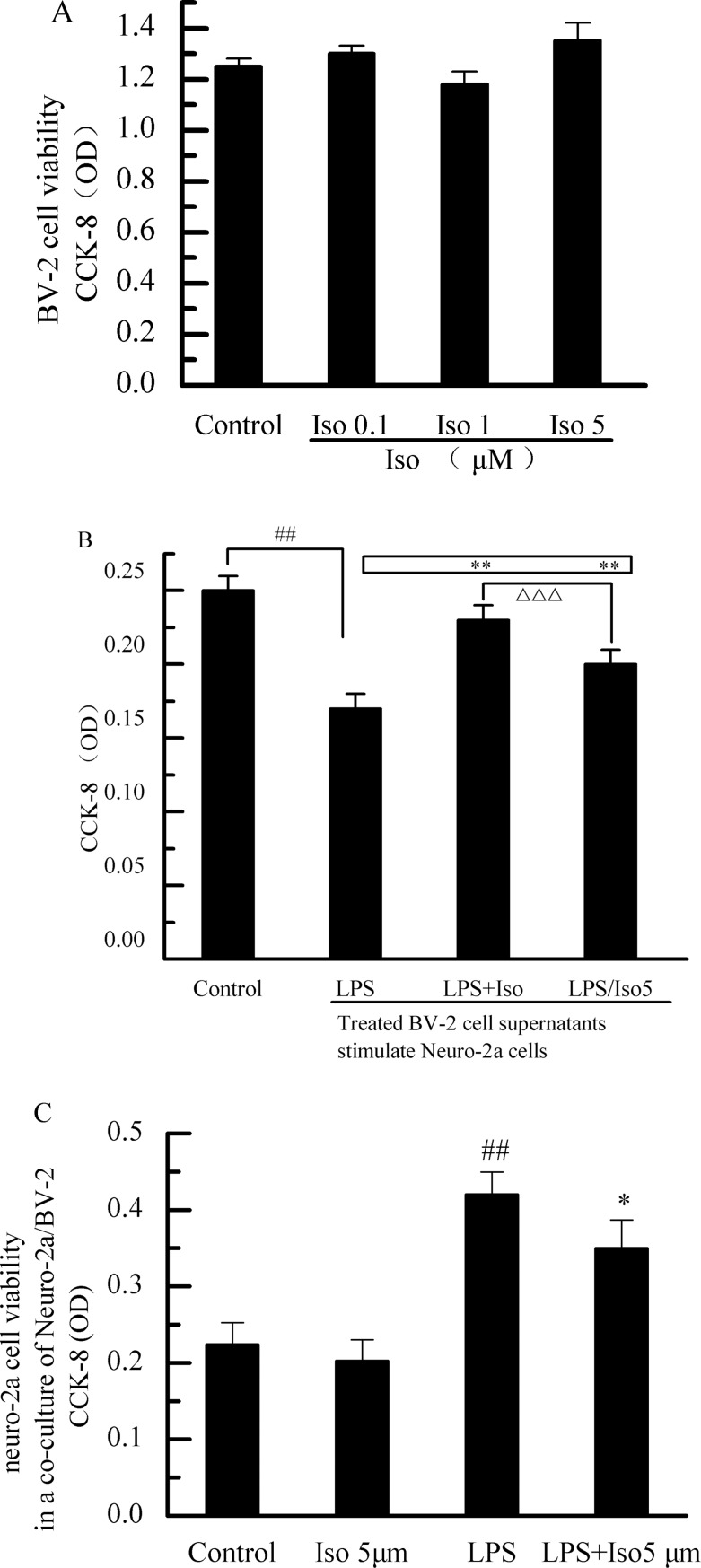
Effects of isobavachalcone treatment on BV-2 cells and Neuro-2a cells. A: Isobavachalcone (Iso) caused no significant cytotoxicity to BV-2 cells. B: The effect of isobavachalcone treated BV-2 cells supernatants on Neuro-2a cells. BV-2 cells CM was treated by LPS-treated together with isobavachalcone (LPS+Iso group), exerting a protective effect on Neuro-2a cells. And such protective effect was stronger than that of the directly treatment with isobavachalcone plusing the supernatant from LPS-treated BV-2 cells (LPS/Iso group). Means: Control (DMEM basic culture medium), LPS (supernatant from LPS treated BV-2 cells), LPS+Iso (supernatant from LPS plus isobavachalcone treated BV-2 cells), LPS/Iso (isobavachalcone (final concentration 5μM) dissolved in supernatant from LPS treated BV-2 cells). C: Isobavachalcone decreased microglial-induced neuro-2a death in a co-culture system. Bars indicated the mean±SD of three independent experiments. ## p<0.01 in comparison to control group. * p<0.05, ** p<0.01 in comparison to LPS group. △△△ p<0.001 in comparison to LPS+Iso group (supernatant from LPS plus isobavachalcone treated BV-2 cells).

Isobavachalcone did not cause a significant cytotoxicity to BV-2 cells (Fig 6A), whereas the supernatant of the conditioned medium for LPS-treated BV-2 cells (LPS group) caused a obviously cytotoxicity to Neuro-2a cells (compared with control group), indicating that the inflammatory cytokines of microglia induced by LPS exerted the cytotoxic effects on Neuro-2a cells. Compared with LPS group, isobavachalcone directly antagonized the cytotoxic effect of LPS-treated BV-2 cells CM on Neuro-2a cells. However, BV-2 cells CM was treated by LPS-treated together with isobavachalcone (LPS+Iso group), exerting a protective effect on Neuro-2a cells. And such protective effect was stronger than that of the directly treatment with isobavachalcone plusing the supernatant from LPS-treated BV-2 cells (LPS/Iso group) (Fig 6B). Moreover, as shown in Fig 6C, we found that isobavachalcone decreased microglial-induced neuro-2a death in a co-culture system.

## Discussion

Microglia, a type of immune cells in central nervous system, are sensitive to external stimuli from the external environment. Under the pathological conditions, such as cerebral ischemia, neurodegenerative diseases, infection, or changes of microenvironmental factors may rapidly activate microglia, thus directly injuring neurons or causing other secondary injuries [9], but the role of microglia in neurodegenerative diseases is still controversial. Some researchers propose that activated microglia may reduce neuron damage and improve tissue repair ability. However, a good amount of contrary evidence shows that activated microglia may actually aggravate nerve damage by producing an excess of inflammatory cytokines, reactive oxygen species, matrix metalloproteinase, and chemokines. Actually, it has been confirmed that inflammatory reactions serve as a double-edged sword in many pathological conditions. It is found that non-steroidal anti-inflammatory drugs [10], tetracycline antibiotic minocycline [11], and naloxone [12] play neuroprotective roles by regulating microglial inflammatory reactions. However, when using those anti-inflammatory drugs to treat certain microglia-mediated nerve inflammation diseases, close attention must be paid to the therapeutic window, lowest effective dose, toxic dose and other indexes of these drugs so that their neuroprotective effects will not be affected and the maximum efficacy can be exerted.

PD is a common CNS degenerative disease with reduced motor ability, muscle rigidity, and tremor as the primary symptoms. The main pathologies of PD are the degeneration and loss of dopaminergic neurons in the substantia nigra and corpus striatum, that sharply reduce the levels of the neurotransmitter DA in the corpus striatum, and the formation of eosinophilic lewy bodies (LB) in cells. According to existing studies, neuroinflammation is a major pathological mechanism of PD, and also is a main target for PD treatment [13, 14]. The autopsy results of PD patients showed that the degenerated neurons were surrounded by many activated microglia, and the same result was also obtained in PD animal models induced by MPTP, 6-OHDA, etc. In this study, neurons were severely injured and accompanied with extensively activated microglia in the substantia nigra and corpus striatum of MPTP-treated mouse, which was consistent with the study of V. Hugh Perry [15]. We also found that a large amount of free radicals and inflammatory cytokines were produced in the brain of PD mouse, and isobavachalcone could decrease the inflammatory factors in both of the PD mouse and BV-2 cells. Moreover, isobavachalcone inhibited microglial activation induced by LPS and alleviated the cytotoxicity of LPS to neuroblastoma Neuro-2a cells. However, isobavachalcone had no influence on the oxidative stress factor in the brain of PD mouse, which was inconsistent with the results in BV-2 cells, and the reason that makes the functional disparity between *in vivo* and *in vitro* need to be further studied.

NF-κB is an important transcription factor that plays vital roles in cell growth and proliferation [16, 17], which can regulate the inflammation by influencing the expressions of its downstream genes, such as TNF-α, IL-1β, IL-6, and IL-8. We confirmed that isobavachalcone mediated the nuclear transfer of transcription factor NF-κB's p65 subunit, and then further regulated the oxidative stress and the expression of inflammatory factors at both mRNA and protein levels.

At present, most studies on the neuroprotective effects of *Fructus psoraleae* have been performed by animal experiments. But, the study on molecular mechanism of isobavachalcone-induced neuroprotective effect is still lack. In this study, we found that the isobavachalcone protected neurons mainly by inhibiting microglia activation for the first time. Assays of its stimulus to BV-2 cells confirmed that isobavachalcone not only acted directly as a neuroprotective agent, but also provided indirect neuroprotective effects by inhibiting microglia activation. These results show that the monomer chemical substance isobavachalcone extracted from *Fructus psoraleae* is a very promising anti-neuroinflammatory drug with great clinical potential against PD, and also suggestting that more effective and robust anti-PD drugs may be developed by modifying the chemical structure of isobavachalcone.

## Supporting Information

S1 Table The residence time of mice on Rota-rod. The duration for a mouse to stay on the rod rolling at 10 rpm. The results were presented as the mean±SD of three independent experiments (n = 6).
(XLS)

S2 Table The quantitative analysis of microglia, astrocytes and neurons. Isobavachalcone (Iso, 50 mg/kg) could inhibit either the elevated levels of Iba-1 and GFAP, or the necrosis of neurons.
(XLS)

S3 Table The mRNA transcription levels of BV-2 cells. The results were presented as the mean±SD (% of control) of three independent experiments (n = 6).
(XLS)

S4 Table The mRNA transcription levels of PD model mouse. The results were presented as the mean±SD (% of control) of three independent experiments (n = 6).
(XLS)

S5 Table The determination results of the serum levels of NO, TNF-α and IL-6. Griess method was used to determine NO concentration, the concentrations of TNF-α and IL-6 were determined using ELISA kits. The results were presented as the mean±SD (% of control) of three independent experiments (n = 6).
(XLS)

S6 Table The result of cell viability assay in BV-2 cells. Isobavachalcone (Iso) caused no significant cytotoxicity to BV-2 cells. The results were presented as the mean±SD of three independent experiments (n = 6).
(XLS)

S7 Table Effect of isobavachalcone-treated BV-2 cells supernatant on activity of Neuro-2a cells. Means: Control (DMEM basic culture medium), LPS (supernatant from LPS treated BV-2 cells), LPS+Iso (supernatant from LPS plus isobavachalcone treated BV-2 cells), LPS/Iso (isobavachalcone (final concentration 5μM) dissolved in supernatant from LPS treated BV-2 cells). The results were presented as the mean±SD of three independent experiments (n = 6).
(XLS)

S8 Table The result of cytotoxicity assay in a co-culture of Neuro-2a and BV-2 cells. Isobavachalcone (Iso) decreased microglial-induced neuro-2a death in a co-culture system. The results were presented as the mean±SD of three independent experiments (n = 6).
(XLS)
